# Collagen Fibril Orientation In Vitro: From Formation to Advanced Biomaterial Development

**DOI:** 10.3390/biomimetics10100644

**Published:** 2025-09-24

**Authors:** Yuliya Nashchekina, Alexey Nashchekin

**Affiliations:** 1 Institute of Cytology of the Russian Academy of Sciences, Center of Cell Technologies, Tikhoretsky Pr. 4, St. Petersburg 194064, Russia; 2 Laboratory Characterization of Materials and Structures of Solid State Electronics, Ioffe Institute, Polytekhnicheskaya Str., 26, St. Petersburg 194021, Russia; nashchekin@mail.ioffe.ru

**Keywords:** collagen fibril, orientation, microstructure, hierarchical organization, self-assembly, tissue engineering

## Abstract

Extracellular matrix proteins have a complex assembly in tissue and it is believed that not only the chemical structure, but also their location, plays an important role in cellular functions. Collagen is one of the main components of the extracellular matrix and the oriented arrangement of collagen fibrils in tissues such as bone, cartilage, tendons, and cornea has a significant impact on various tissue functions. In the body, the orientation of extracellular matrix proteins is determined by cells. Oriented collagen fibrils can not only promote directed cell migration, but also stimulate cells to secrete an extracellular matrix with an oriented structure. However, the creation of collagen fibrils with an oriented structure in vitro is still associated with a number of limitations. Such limitations are primarily because the mechanisms regulating cellular functions in the orientation of extracellular matrix proteins, including collagen, are still unknown. Currently, only physical ways of organizing collagen fibrils in a certain direction are known. We hope that the description of the orientation of collagen fibrils in this review will allow readers to better understand the processes that occur with molecules. The study of methods and conditions for obtaining oriented collagen fibrils can help to obtain tissue biomimetic materials with complex properties identical to native tissues. Therefore, we discuss here various methods and conditions for obtaining oriented collagen fibrils in vitro using mechanical, electric, magnetic, and other fields. The prospects of application in tissue engineering and scientific problems of oriented collagen fibrils are also described.

## 1. Introduction

Biological tissues consist of cells surrounded by extracellular matrix proteins, which are complex of macromolecules, including proteins, glycoproteins, proteoglycans and glycosaminoglycans [1,2]. The components of extracellular matrix play a central role as active regulators of cell functions [3,4]. The interaction between cells and extracellular matrix proteins includes the remodeling of the extracellular matrix by the cells during physiological conditions through the secretion of quite a lot of components of extracellular matrix [5]. Cells react to any change in the structure of the extracellular matrix, and at the same time, a violation of the functional activity of cells leads to a change in their ability to synthesize and structure the extracellular matrix [2]. Collagen is one of the main proteins among the extracellular matrix proteins. Its content in the body reaches 30% of the total amount of all proteins [6]. There are more than 20 different types of collagen, with different functions [7]. In the human body, approximately 90% of collagen exists in the form of fibrils. Not all types of collagen can form fibrils. Types I, II, III, V, and XI collagens are fibrillating. Type I collagen is the most common [8,9]. Type I collagen is the first protein that has been isolated from native tissue. It is the main structural element of the main connective tissues, such as the dermis, bones, tendons, ligaments, and cornea and occupies approximately 70% of the total amount of collagen that is in the body [10]. In vivo, collagen fibrils can assemble into fibers, and the fibers in different tissues are intertwined into specific patterns. For example, the fibers are arranged in parallel in tendons [11], concentric rings in a long bone [12], and also in the cornea [13].

The widespread use of collagen in various tissues has led to the active use collagen as a material for the formation of various kinds of scaffolds for cell cultivation, followed by tissues transplantation [14,15].

Being the main component of connective tissue, type I collagen is not only the main structural element, but also performs a number of regulatory functions. It also has low immunogenicity (i.e., it does not provoke a positive immune response of the body) and weak antigenicity (i.e., it does not interact with antibodies) [16]. Due to its unique biological structure and biochemical functions, collagen is widely used in the formation of scaffolds for tissue engineering and regenerative medicine [17]. Various shapes and sizes of scaffolds are formed of collagen in the form of films, sponges, gels, and tubes [17,18]. Collagen-based scaffolds have been used for cell culture and transplantation since the mid-20th century, but the collagen fibers in such scaffolds were not oriented [19,20].

**Figure 1:**
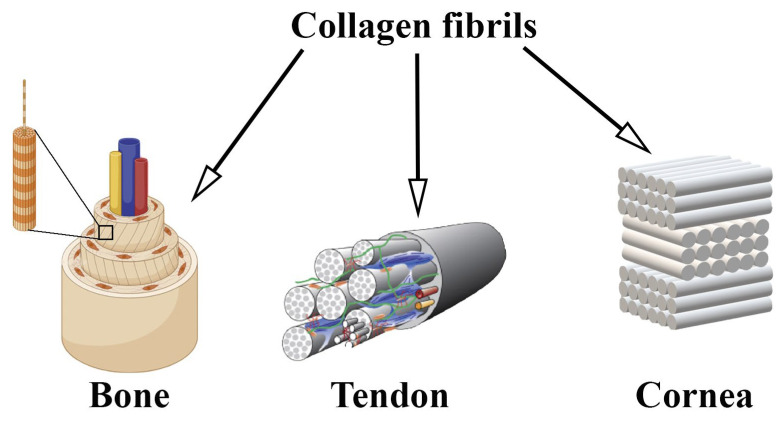
Hierarchical organization of type I collagen in, bone, tendon and cornea. Reprinted from Ref. [29] with permission (Tendon).

Tissue engineering, which focuses on the regeneration and restoration of damaged tissues and actively uses collagen-based scaffolds for culturing and transplanting cells of various tissue origins, still faces the problem of restoring tissue functions [21]. New knowledge about the structure of the extracellular matrix and the mechanisms of tissue regeneration allowed scientists to conclude that collagen scaffolds with an oriented structure play an important role in cell viability in vitro and tissue regeneration. [22,23]. In 2011, Caliari noted that a number of studies have demonstrated successful tissue regeneration using scaffolds with an oriented structure [24]. It has been shown that specific orientation of scaffolds for regeneration of such tissues as peripheral nerves, myocardium, and tendons promotes their rapid regeneration, in addition, oriented fibers stimulate such cellular processes as migration [25,26] and differentiation [27]. When cultured on a collagen scaffold, cells react not only to the chemical structure of the scaffold, but also to its structure, which should repeat the native structure and location of collagen fibrils [28]. In all tissues of the body, type I collagen has a certain multilevel hierarchical structure. Each structural level performs certain biological functions [10]. The orientation of collagen fibrils plays an important role in this hierarchy. In some tissues, such as the skin, collagen fibrils are arranged in a chaotic manner in the extracellular matrix (Figure 1), in other tissues, such as bone, tendons, or cornea, collagen fibrils are oriented parallel to each other. Both collagen isolation and scaffold manufacturing affect the cellular response and the overall biological activity of scaffolds in vivo (Figure 1) [29].

In this review, we will look at the structure of type I collagen ideas development, starting with early works and ending with modern relevant theories. It should be noted that modern theories about the structure of type I collagen do not contradict the theories that were proposed more than fifty years ago. Despite the fact that, in the body, the extracellular matrix is structured by cells during the synthesis of individual molecules, in vitro it is still impossible to “force” cells to orient collagen in the right direction. Currently, the only way to orient collagens in a certain direction is through physical methods of exposure. Therefore, the choice of literature sources in the review is primarily due to data on the physical effects on collagen molecules. It is shown that it is physical factors such as electrical, mechanical, and magnetic that assemble the collagen molecules in the native orientation. Despite the undoubted relevance and practical significance of this topic, the number of publications over the past decades does not seem excessive. We believe that the systematization in one review of physical approaches to the orientation of collagen molecules will allow the authors to choose the optimal method for creating scaffolds for cultivating a certain type of c cells for their further transplantation into a damaged organ. Thus, the purpose of this review is to describe the physical methods of orientation of collagen molecules to obtain skeletons with a structure similar in structure to native tissue.

## 2. Structure of Collagen Type I

The single collagen fibril show a peculiar hierarchical structure [30]. For type I collagen, four different structural levels can be distinguished as follows: primary at the atomic/submolecular scale, secondary and tertiary levels at the molecular scale, and quaternary levels at the supramolecular scale [2].

The primary structure of collagen is an amino acid sequence in polypeptide chains called α-chains. Type I collagen is a heterotrimer consisting of two identical α1 chains and one α2 chain. The α1- and α2-chains differ in amino acid sequence [28].

The collagen molecule is a repetition of the Gly-X-Y triplet, where the amino acid glycine (Gly) occupies every third position in the peptide sequence of a single chain. The X and Y positions are often occupied by proline (Pro) and hydroxyproline (Hyp), respectively [31]. In particular, it is reported that the Gly-Pro-Hyp triplet is unique specifically for collagen molecules and occurs in this form with a frequency of about 12%, while 44% of triplets are in the form of Gly-X-Hyp and Gly-Pro-Y, and the remaining 44% in total in Gly-X-Y [2,32].

A sufficiently high content of the amino acid glycine in the collagen molecule, which reaches 30%, is important for the stabilization of the triple helix domain, since glycine is involved in the formation of inter-chain hydrogen bonds. These hydrogen bonds are formed both between the main NH group of glycine and the main C=O group of the residue at position X [33,34]. The presence of glycine in every third position of this triplet allows for a fairly dense packing of collagen molecules [35].

Proline and hydroxyproline stabilize the triple helix, as well as prevent rotation around the C–N bond and, due to this, stiffen the α-chains [31,32]. Hydroxyproline is a marker for the detection and quantification of collagen in tissues [36]. Collagen contains many proline residues, which have a significant number of cis-peptide bonds. Cis-trans isomerization also explains the experimentally observed hysteresis of collagen unfolding/refolding transitions in vitro [37].

The secondary structure of collagen is represented by the left-sided spiral formation of α-chains. Moreover, as these three chains wind together forming a triple helix, each individual left-handed chain adopts a right-handed superhelix. [38,39,40]. Recent work has shown that with molecular dynamics, the number of hydrogen bonds calculated within collagen is directly correlated to the experimental measurement of triple-helical quality [41]. Triple helix (collagen molecular) contains two short non-helical regions at the ends of the molecule at the amino (N–), and at the carboxy (C–) ends. These regions are known as the telopeptides. Amino and carboxy chains have a size of about 9–26 residues and, unlike the main chain, do not have a repeating Gly-X-Y structure [42].

The triple-helical conformation has two models [2,33,43]. One of the models was proposed by Rich and Crick [33]. This model is based on studies of fiber diffraction on native-type I collagen and takes into account a single inter-chain hydrogen bond per triplet and a 10-fold spiral symmetry with a step of 10/3. Later, another model was proposed, which is based on the crystallographic analysis of proline-rich collagen peptides. Unlike the first model, the second one demonstrates 7-fold spiral symmetry with a step of 7/2. A unified theory that combines the results of the two theories postulates that the collagen molecule has a 10-fold spiral symmetry with a step of 10/3 in proline-poor regions and a 7-fold spiral symmetry with a step of 7/2 in proline-rich regions [44].

In native form, in the body, collagen molecules form fibrils. The molecules in collagen fibrils are quasi-hexagonally packed and super-twisted in a right-handed structure along the longitudinal axis of the fibril [43]. The nucleation and growth of collagen fibrils can occur in a minimal system of purified collagen molecules in warm neutral buffer and indicate an intrinsic tendency for self-assembly. Heating a cold solution of collagen in acetic acid followed by neutralization results in the accumulation of early collagen fibrils with smoothly tapering tips when less than 1% of collagen has assembled. These early fibrils can readily fuse as their concentration increases, a process that promotes an increase in fibril size, resulting in a wide range of diameters in the final gel, typically 20–80 nm [30]. The pathway of fibril assembly critically depends on both the integrity of the telopeptides [45] and the order of heating and neutralization of the initial cold acidic collagen solution: neutralization followed by heating results in the accumulation of thin strands in the early stages of fibril assembly rather than short fibrils with pointed ends [30].

Self-assembly of collagen molecules is a gradual process [42,46], which is influenced by factors such as temperature, pH, ionic strength, and initial concentration [47,48,49]. It is worth noting that the formation of the banded pattern (D-band) of collagen fibrils is associated with the presence of potassium ions [50]. The presence of this D-band pattern is due to the checkerboard arrangement of collagen molecules within the fibril, resulting in a repeating pattern of gaps/overlaps [51,52]. The mechanism of D-band formation remains an open question, and competing theories offer different views on the nucleation and growth of collagen fibrils. One model proposed by Birk and Kadler proposes that individual collagen fibrils nucleate in fibripositors on cells and grow and fuse axially [46,53]. This fibril fusion accounts for the formation of D-band. A second model views collagen as a liquid crystal. Giraud-Guillet et al. proposed that collagen precursors (procollagen or tropocollagen) are pre-aligned in concentrated local environments, which promotes alignment, waviness, and twisting in the collagen fibril packing [54,55]. This model provides a simple physical explanation for the structural organization of collagen in collective tissues and suggests the intriguing possibility that the spacing between D-band is synchronized by liquid crystal alignment. Although aspects of this model are compelling, liquid crystallinity of collagen has not been demonstrated directly in vivo.

Self-assembly of collagen molecules into fibrils under the influence of external factors such as temperature, pH, ionic strength, and initial concentration is a fairly common model describing the hierarchy of collagen in the body. However, there is another hierarchical model that shows that between the collagen molecule (triple helix) and the fibril there is an intermediate structural unit called a microfibril [51]. This structural model was proposed after obtaining radiograph results of diffraction of native collagen. According to this model, the “Microfibril” is considered to be the basic building block of collagen fibril. Based on theoretical and experimental results, the authors proposed that the repeating arrangement of pentameric collagen molecules is sequentially self-satisfied with respect to the collagen packing lattice and can be accurately described as a microfibril consisting of five one-dimensional step-like, folded collagen molecules with right-handed supercoiling. This theory is consistent with the microfibril model that Hodge and Petrushka proposed earlier [56,57]. Hodge and Petruska assumed that collagen fibrils are a two-dimensional stack of five collagen molecules. These molecules are aligned parallel to each other with a step D-band of about 67 nm. Two other important parameters of the native-type fibril, besides D, also are predicted to match integral multiples of the major turn of the triple helix. One of these is the “gap” or “hole” between nearest nonoverlapping macromolecules in the fibril axis. The other parameter of the native-type fibril is the end overlap between nearest macromolecules displaced axially relative to one another by 4 D. It is this overlap that is responsible for the endjunction in both native-type fibrils and the ordered fibrous aggregates of period 4 D known as F-SLS. Much later, with the help of transmission electron microscopy, it revealed a characteristic D-periodicity of fibrils, which is an axial displacement and the sum of the areas of rupture and overlap between collagen molecules [2]. The length of the collagen molecule is about 300 nm, and a gap with a low electron density is formed between the longitudinally aligned molecules [31,32]. When analyzed by transmission electron microscopy, the regions of gaps between molecules are visualized as darker.

The size of type I collagen fibrils can reach more than 500 microns in length and 500 nm in diameter [2,31,32]. The formation of fibrils in vivo is a process of spontaneous self-assembly [58]. As a result of this assembly, hydrophobic and electrostatic interactions occur between neighboring molecules in order to reduce the surface area of the formed collagen fibrils with their final volume [32,42]. In the body, not only spontaneous assembly is involved in the formation of fibrils, but cells also regulate this process [42]. Since each tissue has its own structure and performs certain functions, in each type of tissue, the process of assembling fibrils occurs according to a specific mechanism that has yet to be studied.

There is evidence that C-terminal telopeptides are involved in the initiation of fibrillogenesis and accelerate the process of fibril assembly [31], although triple helices devoid of telopeptides can also assemble into fibrils [59]. Neuropeptides have also been shown to play an important role in the mechanical stabilization of fibrils [60]. This property is because the terminal telopeptides contain sites for both intramolecular and intermolecular cross-linking.

The stabilization of the molecular structure is provided not only by terminal telopeptides, but also by water molecules that surround each triple helix and form a hydration cylinder [43,61]. The degree of glycosylation, i.e., galactosylation and glucosyl-galactosylation of hydroxylysine residues, affects the diameter of single triple helices, and intermolecular intercenter distances depend on the diameter of these single triple helices. Thus, by changing the degree of glycosylation, the organization of fibrils can be physiologically regulated. This can be observed in type I corneal collagen [62].

Fibers are formed at the next hierarchical level, where collagen fibrils are interconnected by interfibrillar proteoglycans. The typical packing distance for fibers is 100 nm [31,38]. Proteoglycans bind collagen in certain areas with an interval of 60 nm and form intersecting bridges between neighboring fibrils [61].

Despite the apparent simplicity in the structure of the collagen molecule, it has unique properties due to its amino acid composition and sequence, which determine its unusual properties, different from other biological macromolecules. Thus, the five-membered cycles of proline and oxyproline, which are part of the alpha chain of collagen, provide rigidity and further the ability to form “rigid” fibrils. Glycine, which is every third amino acid in the amino acid chain, allows alpha chains to twist into a spiral and interact with each other. The opposite charge of various amino acids at the ends of the collagen molecule ensures the amphiphilicity of the protein molecules and, thus, it becomes possible to “manipulate” the orientation and movement of such a molecule under the influence of an external electric field. Of course, a sufficiently large size of such a macromolecule is a limitation for its movement in space and on the plane.

## 3. Collagen Fibril Orientation Theory

The collagen molecule has been studied by many research groups for quite a long time. Yet despite the apparent simplicity of the structure, there are still many questions that need to be answered. Due to such structural features as amino acid composition, three-chain structure, and different charges at the ends of the molecule, there are several methods by which the collagen molecule can be “controlled” under the influence of external factors. Of course, only an understanding of the molecular structure of collagen will allow us to select the optimal parameters for the influence of external physical factors that can orient and move the molecule in the desired direction.

The structure of collagen fibrils and their properties are influenced by such factors as protein concentration, temperature, pH, and ionic strength of the solution [15,42,46]. Also, the formation of a collagen structure with a D-periodicity is conditioned by the presence of potassium ions in the solution [50]. Oriented collagen fibrils play an extremely indispensable role in the structure and function of tissues [63]. Many functions of cells such as cell adhesion, migration, proliferation, and metabolism depends on oriented collagen fibrils [64,65]. Oriented collagen fibrils are an important basis for tissue morphology [66,67], mechanical properties [68], and wound healing [69].

**Figure 2:**
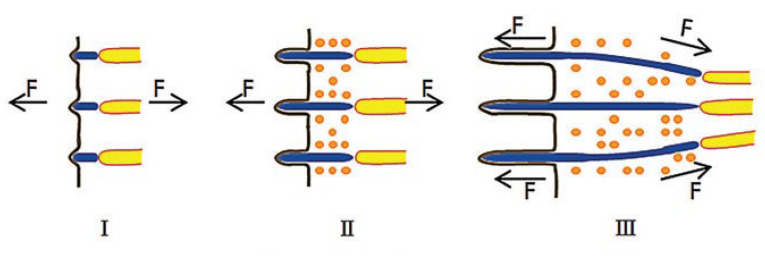
Tendon growth model. (**I**) Collagen assembles on the cell membrane to form microfibers. (**II**) The fibripositors internalize and extend. (**III**) During the growth process, the fibers are slowly pulled out by tension, and the fibers are gradually gathered together by tension. Reprinted from Ref. [65] with permission.

One of the first studies of the oriented collagen fibrils structure was the work of Birk and Trelstad. In 1984, they discovered collagen fibers in sections of the chicken embryo cornea [1]. They believed that oriented fibrils form in the compartments, and the microfibers grow into a dense bundle by lateral fusion of the compartments, as has been demonstrated. In a later work by Canty, it was suggested that oriented fibrils are synthesized in special microtubules [53]. A continuation of this theory in 2013 was the work of Kalson. He believed that the pores exert an internal pulling force on the fibers and interact with the tension of the tissue to make the fibers organized [70]. The patent showed that it is the mechanical load that affects the formation of oriented fibers. He proposed a model according to which tendon growth occurs under a tensile load [71]. There is also an assumption that it is the high concentration of collagen in the solution that contributes to its self-organization into oriented fibrils [65]. A schematic representation of one of the models is shown in Figure 2.

Figure 2 shows a model of the formation and growth of fibers in tendons. According to this model, collagen originates on the cell membrane and is already assembled into microfibers as the amount of collagen monomer increases [65,72]. Currently, there is no unambiguous theory of the orientation of collagen fibrils in vivo. However, several methods of orientation of collagen fibrils under the influence of external factors such as mechanical action, magnetic field, or electric field are presented in the literature.

## 4. Mechanical Environment

The role of mechanical force in the formation and development of collagen structure is still actively studied. A theory of the mechanochemical mechanism of collagen tissue assembly dynamics posits that collagen fibrils are preferentially deposited and grow directly in the path of mechanical force. Under certain conditions, particularly where necessary, mechanical stress stimulates collagen fibril synthesis. This mechanism is called the mechanochemical cause-and-effect relationship between force and structure [73].

Mechanical loading has an important influence on the formation and morphology of tissues. During the vital activity of the body, due to the movement of the limbs during walking, tissues are subjected to various mechanical loads, such as stretching, compression, and shear [74].

In accordance with the loading mode, the method of preparing fiber orientation mediated by shear is mainly divided into fluid displacement controlled by fluid flow and mechanical displacement created by the relative motion of the acting surfaces.

In 2008, the authors applied liquid deposition techniques to obtain oriented fibers [75]. In the experiment, the collagen solution flowed through a miniature rectangular tube. The degree of orientation of the fibers gradually decreased with decreasing flow velocity. An increase in the concentration of the collagen solution also contributes to an increase in collagen orientation. In 2009, it was demonstrated that the self-assembly of collagen is regulated by a fluid shift [76]. The different shear rates created by the fluid flow regulated the process of assembling the collagen monomer on the glass sheet, and the prepared fibers were oriented along the direction of the fluid flow. At different shear rates, the rate of axial growth of fibers is different. As the shear rate decreases, the length of the fibers increases. Oriented fibers obtained by this method do not have a striped pattern. Saeidi, with their coauthor, demonstrated that assembled fibrillar structure did not possess native D-periodicity. Instead, fibrils comprised a collection of generally aligned monomers which were self-assembled to form a fibril-like aggregate. Thus, the shear rate affects the distribution of the collagen monomer and the method provides a certain attachment position of the fiber due to the morphology of the surface and allows the fiber to grow in a certain direction. Subsequently, based on previous fluid displacement experiments, Saeidi corrected the experimental scheme by obtaining orthogonally oriented fibers due to a combination of fluid flow and a circular film. Experiments have shown that the degree of orientation of the fibers varies greatly at different flow rates and rotational speeds. Excessively high rotational speeds prevent the fibers adhering to the substrate, while at lower rotational speeds, the deposited fibers are disorganized.

In 2011, Lai made the assumption that the combined use of a high shear concentration makes it possible to obtain oriented collagen fibrils due to the liquid crystal state of collagen [77]. To obtain such oriented fibrils, he used a special syringe. In 2015, this theory was put into practical use and Hoogenkamp applied the technology of cone extrusion with reverse rotation to produce oriented collagen fibers [78]. In such stretching-mediated experiments, the force can affect both the collagen monomer and the entire oriented collagen fibril. As a result, the directed distribution of collagen monomers occurs, and when exposed to a fibril or fiber, their straightness improves.

In 2001, Wilson formed oriented collagen fibrils by nanolithography using an immersion pen. He drew a line in a certain direction on a gold substrate using a solution of thiolated collagen, which adhered to the probe of an atomic force microscope [79]. During the process, collagen monomers adhere to the substrate and assemble into oriented fibrils. The attachment of collagen monomers to the probe occurs due to cohesion and Coulomb force. Paten used the subunit principle in 2016, when a glass needle was used as a probe, which allowed the collagen solution, including high concentrations, to be pulled in one direction [71]. After immersing in a high-concentration collagen solution, the needle was slowly extracted and a continuous column of liquid formed between the micro needle and the drop. In such a continuous column of liquid, collagen monomers are assembled into oriented fibers. The authors believed that the reason for the orientation of monomers is the hydrolytic effect.

Orientation of collagen is possible also on special flat substrates consisting of lanes for direct adsorption of collagen. Spontaneous adsorption of collagen, carried out on such structured substrates, leads to the accumulation of collagen on hydrophobic tracks with widths from 30 to 90 nm. The dimensions of these tracks are much smaller than the length of collagen molecules (about 300 nm), which leads to their alignment. Due to the self-limitation of proteins during adsorption, this effect can find a new and valuable application for obtaining surfaces with oriented collagen [80]. This method attracts with its simplicity and accessibility. However, such scaffolds’ actual practical application is doubtful due to the difficulty of detaching such structure from the substrate and maintaining its shape. The resulting structured collagen surfaces can serve as a model for studying the adhesion, proliferation, and migration of tissue-specific cells, for which the orientation of the extracellular matrix is an important and necessary parameter.

In 2008, Vogue applied a formed collagen gel as a collagen source, which was affected by cyclic uniaxial stretching [81] as a result of stretching the collagen fibers located along the direction of tension. Vader et al. conducted similar studies later [82]. He showed that an increase in tensile strain leads to a continuous increase in the degree of fiber orientation. They also noted a significant orientation of the fibers along the force line with the maximum degree of orientation near the force line.

Despite the relative simplicity and availability of obtaining oriented collagen fibrils by mechanical loading, extrusion, and the use of microfluidic channels, scaffolds obtained by this method have significant drawbacks [83,84]. It is impossible to obtain scaffolds with high packing density and elastic deformation. Thus, the final products obtained in this way are, as a rule, small in size, and can only be used as models for studying the behavior of cells in vitro, but not as tissue engineering structures intended for transplantation into organisms.

## 5. Magnetic Orientation

The use of a strong magnetic field is one of the promising methods for the formation of oriented scaffolds [85]. The principle of orientation is that magnetic anisotropy contributes to the generation of a magnetization force torque, which rotates the crystal to adopt a stable orientation and reduce the magnetization energy [86]. Due to the magnetic anisotropy of collagen molecules [87], collagen fibrils can orient themselves under the influence of a magnetic field [88]. The use of a magnetic field is another way to obtain scaffolds with oriented collagen fibrils. It was also reported that collagen fibrils are oriented in planes normal to the direction of the applied magnetic field [89]. It is possible to increase the effect of the magnetic field by adding magnetic beads to the collagen solution.

In the case of a strong magnetic field, collagen molecules line up perpendicular to the field, since each collagen molecule has a negative diamagnetic susceptibility. In 2007, Torbet created oriented hydrogels, and fibrillation was initiated in a collagen solution that was placed under the action of a magnetic field with an induction of 70,000 G [25]. The use of magnetic fields 3–4 times weaker allows the addition of magnetic particles to collagen solutions.

Guo used microscopic iron oxide beads with an approximate diameter of 1.5 to 2.5 microns modified with streptavidin, carboxyl, or amine [88]. This solution was exposed to a magnetic field with induction up to 2 G with simultaneous heat treatment to induce fibrillation. It is believed that the reason for orientation is the movement of modified balls with fibrils stuck to them along the lines of force to the poles of the magnet during gelation. Authors in [90] used magnetic nanoparticles with a diameter of 50 nm and 100 nm. They induced fibrillation in solution and applied an external magnetic field by induction from 162 G to 2110 G. The application of a magnetic field during the gelation period leads to the aggregation of particles along the magnetic field lines and the orientation of collagen.

Magnetic iron oxide nanoparticles can be added to the collagen gel to remote control orientation [91,92,93,94]. Segmented collagen I hydrogels with fiber-like structures organized in parallel with the orientation of the particles were formed using magnetic iron oxide nanoparticles. Such hydrogels exhibit anisotropy. The orientation of scaffolds and cells cultured in them with the help of a magnetic field was studied with the directed growth of neurons in collagen gels [90,95]. In the work of Wright and coauthors, the relevance of this approach was demonstrated during creating tendons in which the correct orientation of collagen fibers in the extracellular matrix plays an important role [94].

It was shown that adult stem cells line up along oriented collagen fibers. Thus, the alignment of type I collagen in the magnetic field provides unidirectional isotropic organization.

Despite the apparent simplicity and accessibility of the magnetic method, it also has a number of limitations. The main limitation, in contrast to the mechanical forces orientation, is the need for superconducting magnets of the Tesla order, since the collagen molecule itself has a low diamagnetic constant [96].

## 6. Electrochemical Orientation

Cheng et al., in 2008 [97], proposed an electrochemical method to orient collagen fiber (ELAC, electrochemically aligned collagen bundle). The procedure is as follows: a dialyzed collagen solution is placed between two electrodes and under the influence of electric current on the electrodes, and the following reactions occur:

at the anode: 2H_2_O − 4e^−^ → 4H^+^ + O_2_

at the cathode: 4H_2_O + 4e^−^ → 4OH^−^ + [2H]_2_

After generation of hydrogen and hydroxyl groups, a pH gradient forms between the electrodes. The collagen molecule is an ampholite, that is, there are both acidic and basic groups in it. Therefore, near the electrodes, the molecule receives the same charge with the electrodes. Under the action of electrostatic forces, the molecule is repelled from the similarly charged electrode and concentrated at the isoelectric point of collagen at pH = 8.2 (for type I collagen). So, the concentrated molecules are oriented, with the formation of an oblong fibrous structure. After the electrochemical reaction, the sample is treated with a buffer to initiate fibrillation.

Gurkan showed, in 2010, the absence of cytotoxicity in these matrices, as well as an increase in the ability to migrate tendon fibroblast and mesenchymal stem cells of the bone marrow to electrochemically oriented collagen compared to undirected fiber. Kishore et al. showed the induction of technogenic differentiation in mesenchymal stem cells cultured on electrochemically oriented collagen [98].

In addition to magnetic anisotropy, the collagen molecule also has electrical anisotropy. In 2010, the Fia received oriented collagen fibrils after exposure to various electric fields [99,100]. Three types of electric fields were used to orient collagen fibrils: direct electric field, pulsating electric field, and compound electric field. In this study, authors used a composite material based on collagen gel and hydroxyapatite. The composite material was previously freeze-dried. As a result of the experiment, it was shown that it is the pulsed electric field that has the best effect in the formation of oriented collagen fibrils.

The electrochemical orientation method is used to form collagen fibers for tendons [97,101], cartilage [102], nerves [103], and vascular tissue engineering [104]. The electrochemical method of collagen orientation allows for the formation of complex tissue-like structures [105], and was used to form an artificial cornea. However, such a cornea still requires modification, since its thickness exceeds several times the thickness of the native cornea.

## 7. Biomedical Application of Oriented Collagen Fibrils

Collagen is a versatile biological material that is in great demand in many areas of regenerative medicine and biotechnology. The greatest demand, of course, relates to tissue engineering (Table 1). Cultivation and transplantation of cells of various tissue origins on collagen scaffolds demonstrated positive results in tissue repair. However, in most cases, the authors present positive results, while the imperfection of the scaffold structure, namely, the discrepancy between the created structural organization of proteins compared to native tissues, significantly reduces the efficiency of regeneration. As noted earlier, in some tissues, collagen has directed regeneration, and as shown by the authors of many studies, it is the directed orientation of collagen fibrils that promotes cell differentiation in a certain tissue-specific direction [106].

For example, to understand the molecular mechanisms that regulate the proliferation and differentiation of cardiac myocytes, scaffolds based on mechanically oriented collagen were developed [107]. Such a model will help develop new therapies for myocardial recovery. The key component of this model is a new three-dimensional tubular scaffold constructed from oriented type I collagen fibrils. In this model, embryonic ventricular myocytes undergo a transition from a hyperplastic to a resting phenotype, exhibit significant myofibrillogenesis, and form critical intercellular connections. In addition, fetal cardiac myocytes grown on an oriented scaffold have a flattened phenotype that closely resembles neonatal ventricular myocytes in vivo.

**Figure 3:**
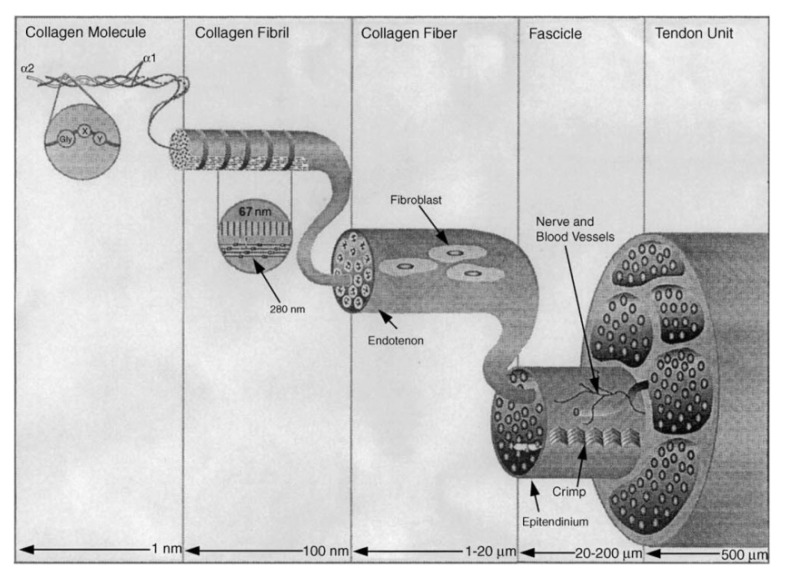
Structural hierarchy in a tendon. Diagram illustrating the relationship between collagen molecules, fibrils, fibers, fascicles, and tendon units. Reprinted from Ref. [66] with permission.

The orientation of collagen fibrils plays an important role in ligaments and tendons. In these tissues, type I collagen molecules are anisotropically oriented in the direction along the dominant physiological load [108]. This parallel orientation provides strength to these tissues, as well as in a physiologically healthy state to withstand prolonged stress. At the moment, there is a theory that in vivo orientation of collagen fibrils in ligaments and tendons occurs due to mechanical loading [66]. After analyzing the experimental data of these authors, we concluded that in vivo collagen self-assembly occurs by deposition of thin fibrils in depressions within the cell membrane. These thin fibrils later increase in length and width due to the lateral fusion of intermediates (Figure 3).

While in vitro collagen self-assembly occurs both at linear and lateral stages of growth, in the absence of cellular control and enzymatic degradation of propeptides, the growth mechanism is altered and the fibrils have an irregular cross section [109]. Proper fibril development in vitro and in vivo determines their final structural and physical properties [110]. Collagen fibrils are long and roughly cylindrical, and are composed of multiple long, chiral, semi-flexible collagen molecules [111]. Although the collagen molecules are nearly parallel to the fibril axis, they exhibit a tilt relative to the axis that is visible both at the fibril surface and within the fibril and this is particularly pronounced in corneal fibrils [13,112]. Corneal fibrils also exhibit tight radius control, which is essential for corneal transparency [113]. In contrast, tendons exhibit an exceptionally wide range of fibril radii 10–13 and a much lower molecular tilt [114]. Enzymatic cross-links, catalyzed by the enzyme lysyl oxidase (LOX), form between collagen molecules during fibril formation [115]. These cross-links are covalent bonds and contribute significantly to tissue elasticity [116].

The results of mechanical studies show that before the start of movement, the mechanical response of the tendon to the load is dominated by the viscous sliding of collagen fibrils. On the contrary, after birth, when locomotion begins, the mechanical response is dominated by the elastic stretching of cross-linked collagen molecules. Thus, it has been shown that the orientation of collagen fibrils in vivo is provided by many factors, and the presence of a mechanical load is the determining parameter that ensures the strength and elasticity of the tendons.

The orientation of fibrils plays the most important functional role in bone tissue [117]. It is known that in the process of remodeling, the cortical bone is continuously rebuilt in response to mechanical loads in order to provide sufficient mechanical strength of the bone tissue at all hierarchical levels [118]. While bones are predominantly oriented parallel to the longitudinal axis of the bone, the orientation of collagen fibers within individual lamellae can vary significantly, leading to a number of proposed models over the years [119,120,121]. On the one hand, collagen in bone tissue is oriented, but its orientation has different directions, usually associated with the direction of the load axis. In the process of forming collagen fibers in mammalian bone tissue, mechanical load affects the orientation, and at the same time, oriented collagen fibrils have increased mechanical properties compared to non-oriented collagen fibrils. The cause and impetus that drive the formation of collagen—a mineral composite—in bone tissue remains poorly understood. Studies of the organic fraction of glass sponges (Hexactinellida) have revealed predominantly hydroxylated fibrillar collagen containing an unusual motif [Gly–3Hyp–4Hyp]. The authors suggest that this motif is predisposed to silica precipitation and represents a new template for biosilicification in nature [122]. A study of spongin, an ancient biocomposite, has demonstrated a complex interaction between collagen structures and cross-links [123].

The mineral units in bone are needle-shaped and merge laterally to form platelets. These platelets are organized into stacks of roughly parallel platelets. These platelet stacks coalesce into larger aggregates that exceed the lateral dimensions of the collagen fibrils and form a continuous cross-fibrillar mineralization that spans adjacent fibrils [124]. Most of the extrafibrillar crystals are also oriented parallel to the collagen fibers, although some variations in alignment have been noted in the non-uniform areas of the bone. The mechanism of collagen mineralization is being actively studied and is currently [124].

To improve mechanical properties, collagen fibers are reinforced with bone mineral particles, forming a bound composite material in which the long axis of the mineral forms a structure with collagen fibers that forms bones with special mechanical properties [125]. In a healthy state, the bone is able to withstand complex physiological loads due to the mechanisms of resistance to destruction [126]. Many factors affect the fracture toughness of human cortical bone, which can become more prone to fracture due to aging, disease, or other environmental conditions. The distribution of bone mineral density, the quality of the minerals, and the accumulation of microcracks determine the resistance of bone to fracture [127]. This extends to the organic part of the bone matrix, where changes in collagen quality have been shown to adversely affect mechanical stability [128]. It is shown that the orientation of collagen fibers is considered as a factor affecting the ability of the bone to resist fractures [129]. It has been suggested that collagen fiber structure is an excellent indicator of cortical bone strength [130]. The dependence of the population density of various types of bones under certain loading regimes suggests that collagen fibers align in accordance with certain deformation regimes and perform various mechanical functions [131].

The literature contains numerous studies on the formation and use of collagen-inorganic composite matrices with various structural and functional properties. Understanding the structure and mechanisms of interaction between collagen molecules, as well as with other components of the organic and inorganic components of bone tissue, will enable the in vitro formation of a composite with functional properties similar to bone tissue [132]. Modern literature data has demonstrated that when studying native bone tissue, it was shown that hydroxyapatite molecules interact with collagen through osteocalcin [133]. This osteocalcin is a mediator molecule for the formation and growth of hydroxyapatite.

No other tissue in the body is more dependent on the composition and organization of extracellular matrix components for normal structure and function than the corneal stroma. The orientation of collagen fibrils, lamellae, and keratocytes is necessary for adults to maintain stromal function. The orientation of collagen fibrils depends on the regulated interaction of the components of the extracellular matrix, which contribute to the achievement of the unique properties of the cornea, namely, transparency, shape, mechanical strength, and vascularity. The precise structural organization of the stroma is a necessary condition for the transparency of the cornea, which depends on the regular packing of homogeneous collagen layers of small diameter fibrils [134]. Fibrils are evenly distributed at an equal distance from each other [10,135]. The structure and organization of collagen fibrils are the most distinctive features of the corneal stroma. The predominant type of collagen that forms fibrils in the corneal stroma is collagen I. However, the structure of the corneal stroma and the structure of the extracellular matrix, in general, is not determined by the type of collagen present. Collagen I is the predominant collagen in striated collagen fibrils, which have a wide range of tissue-specific structures and organizations.

Traditionally, collagen has been regarded as structural scaffolds for cell cultivation. Recent progress demonstrated that collagen remodeling elicits a myriad of biophysical, biochemical, and cell biological alterations that profoundly affect tumor metabolism, growth, and immunity. These effects exerted through interactions with specific collagen receptors either promote or attenuate cancer development [136].

## 8. Conclusions and Perspectives

This paper briefly discusses the main structural features of collagen, the stages of its formation, as well as methods for obtaining oriented scaffolds from collagen fibrils, and describes the prospects for their use in the restoration of damaged tissue. There are many data on the structure of collagen fibrils in the literature, but the mechanism of self-organization of fibrils among themselves is still insufficiently studied. Moreover, there is still no exact information on how and under the influence of what factors collagen is oriented in vivo. Collagen molecules orient in vitro based on the structure data that researchers currently have. For mechanical orientation, the main parameters of collagen are the viscosity of the collagen solution, which can be changed during the experiment. The magnetic orientation of collagen is possible due to the presence of anisotropy in the collagen molecule. The collagen molecule is an ampholite; that is, both acidic and basic groups are present in it. Thanks to these properties, collagen molecules are able to orient themselves under the influence of an electric field. In this regard, despite the fact that the methods for obtaining oriented matrices from collagen fibrils are diverse, and some of them have achieved ideal results in application, it has not yet been possible to obtain a native structure in vitro.

In future studies, three points should be noted as follows:

1. The methods of obtaining oriented collagen fibrils are different, but it is still a difficult problem to obtain oriented collagen fibrils with a structure similar to the structure of the arrangement of collagen fibrils in native tissue. Therefore, it is still necessary to pay detailed attention to the study of the mechanisms of orientation of collagen fibers.
2. Many factors affecting cell functions have been found, but the mechanism of factors affecting the secretion of directed collagen fibrils needs further study.
3. There are many methods of obtaining collagen scaffolds for targeted regeneration, but the location of collagen fibrils in the scaffolds themselves is rarely studied. In future studies, we should pay more attention to promoting the restoration of the structure and function of the newly formed tissue.

Analyzing the data presented in the literature on the structure of the collagen molecule and its ability to form oriented collagen fibrils, we concluded that the main factors affecting the orientation of collagen in the body are physical activity, since, as has been repeatedly demonstrated, the orientation of collagen fibrils and fibers in tendons and bone tissue is consistent with the direction of physical activity. However, there are certain doubts to what extent this method can be used to orient collagen in vitro, since, as noted above, the “participation” of mechanical loads in the orientation of collagen occurs at the moment the tropocollagen molecule leaves the cell to the surface of the cell membrane. The immature molecules are subjected to mechanical stress and their quantity is strictly dosed by the cells themselves. When trying to orient collagen molecules in vitro, in most cases, it is not possible to obtain oriented structures with great accuracy. The collagen molecules used in the experiment are usually already mature enough to be easily manipulated. We believe that this method of orienting collagen in vitro may be promising under mechanical loads of cells synthesizing collagen. However, to date, it is rather difficult to obtain ready-made scaffolds of sufficient volume by synthesis of collagen only by cells, since the synthesis of collagen by cells outside the body is still very slow.

The method of orienting collagen under the influence of a magnetic field also still has a number of limitations due to the need for exposure to a source with a sufficiently high magnetic field, as well as the introduction of magnetic particles into the scaffold composition, which can subsequently affect the properties of the final scaffold and the viability of the cells cultivated inside it.

We believe that the most promising method for targeting collagen in vitro is the electric field. Despite the fact that the collagen molecule is quite large in length, due to the fact that it is an ampholyte and is charged in an electric field, it is possible to control the speed and direction of its movement, and the mechanism of this process can be controlled by varying the voltage, current strength, and dielectric constant of the medium.

## Figures and Tables

**Table 1:** Summary and comparison between mechanical, magnetic, and electrochemical orientations.

	Fabrication Methods		
	Mechanical Orientation	Magnetic Orientation	Electrochemical Orientation
Orientation processes	The method mediated by shear is mainly divided into fluid displacement controlled by fluid flow and mechanical displacement created by the relative motion of the acting surfaces [74,75].	Due to the magnetic anisotropy of collagen molecule, collagen fibrils can orient themselves under the influence of a magnetic field [87,88].	The collagen molecule is an ampholite and under the action of electrostatic forces, the molecule is repelled from the similarly charged electrode and concentrated at the isoelectric point [97,98].
Adjustable parameters	Flow rates and rotational speeds, concentration of collagen in solution.	Structure and quantity of magnetic particles, magnitude of magnetic field.	Concentration, voltage, current density, and time.
Limitations	Oriented fibers obtained do not have a striped pattern. The final products are small in size. It is impossible to obtain scaffolds with high packing density and elastic deformation.	The main limitation is the need for superconducting magnets of the Tesla order, since the collagen molecule itself has a low diamagnetic constant.	Currently, the largest limiting factor of electrochemical orientation collagen use is its insufficient mechanical strength.

## Data Availability

Not applicable.
